# Ameloblastin in Hertwig’s Epithelial Root Sheath Regulates Tooth Root Formation and Development

**DOI:** 10.1371/journal.pone.0054449

**Published:** 2013-01-23

**Authors:** Naoto Hirose, Atsushi Shimazu, Mineo Watanabe, Kotaro Tanimoto, Souichi Koyota, Toshihiro Sugiyama, Takashi Uchida, Kazuo Tanne

**Affiliations:** 1 Department of Orthodontics, Applied Life Sciences, Hiroshima University Institute of Biomedical & Health Sciences, 1-2-3 Kasumi, Minami-ku, Hiroshima, Japan; 2 Department of Public Oral Health, Integrated Health Sciences, Hiroshima University Institute of Biomedical & Health Sciences, 1-2-3 Kasumi, Minami-ku, Hiroshima, Japan; 3 Department of Oral Biology, Basic Life Science, Hiroshima University Institute of Biomedical & Health Sciences, 1-2-3 Kasumi, Minami-ku, Hiroshima, Japan; 4 Department of Biochemistry, Akita University Graduate School of Medicine, 1-1-1 Hondo, Akita, Japan; The University of Tennessee Health Science Center, United States of America

## Abstract

Tooth root formation begins after the completion of crown morphogenesis. At the end edge of the tooth crown, inner and outer enamel epithelia form Hertwig’s epithelial root sheath (HERS). HERS extends along with dental follicular tissue for root formation. Ameloblastin (AMBN) is an enamel matrix protein secreted by ameloblasts and HERS derived cells. A number of enamel proteins are eliminated in root formation, except for AMBN. AMBN may be related to tooth root formation; however, its role in this process remains unclear. In this study, we found AMBN in the basal portion of HERS of lower first molar in mice, but not at the tip. We designed and synthesized small interfering RNA (siRNA) targeting AMBN based on the mouse sequence. When AMBN siRNA was injected into a prospective mandibular first molar of postnatal day 10 mice, the root became shorter 10 days later. Furthermore, HERS in these mice revealed a multilayered appearance and 5-bromo-2′-deoxyuridine (BrdU) positive cells increased in the outer layers. In vitro experiments, when cells were compared with and without transiently expressing AMBN mRNA, expression of growth suppressor genes such as p21^Cip1^ and p27^Kip1^ was enhanced without AMBN and BrdU incorporation increased. Thus, AMBN may regulate differentiation state of HERS derived cells. Moreover, our results suggest that the expression of AMBN in HERS functions as a trigger for normal root formation.

## Introduction

After the completion of crown morphogenesis, tooth root formation is initiated under a regulatory mechanism with an interaction between inner and outer enamel epithelia, which forms Hertwig’s epithelial root sheath (HERS) [1]–[3]. HERS proliferates downward to the apical region and controls tooth root formation. Epithelial cell rests of Malassez, derived from HERS, are located in periodontal ligament tissues near the developing tooth root and remain in periodontal ligaments throughout life [1], [3]. Root formation is characterized by a series of sequential interactions between HERS and dental follicles. During this process, dental follicular cells are differentiated into odontoblasts. However, HERS cells undergo no calcification and diminish expression of amelogenin and enamelin, except for ameloblastin (AMBN), although HERS cells are derived from the enamel epithelium [1].

AMBN is an enamel matrix protein, also known as sheathelin or amelin, is secreted by ameloblasts, and has the second highest content among all enamel proteins in mature enamel [4]. Immediately after ameloblasts secrete AMBN for enamel formation during crown morphogenesis, AMBN is cleaved into several fragments [5] by its own proteolytic enzymes such as enamelysin (matrix metalloproteinase - 20) and kallikrein-4 after secretion from ameloblasts [6], [7]. Fragments spread and localize in various sites in newly formed enamel [8]. These fragments may have biological activity because AMBN contains the binding domains for calcium [8], [9], fibronectin [10] and heparin [11]. Recent studies indicate that AMBN knockout mice show abnormal enamel structures and ameloblasts fail to adhere to immature enamel layers, which then partially detach from the incisors [11]–[13]. In addition, ameloblasts in these mice lose their ability to maintain normal polarization and exhibit marked proliferation, suggesting that AMBN is required for growth and differentiation of these cells and is necessary to constitute enamel structure.

Although AMBN has generally been believed to be located in ameloblasts, recent studies report that AMBN is not a specific protein in ameloblasts and is expressed in odontoblasts [14], [15], osteoblasts [16], [17] and cementoblasts [18]. AMBN also increased proliferation in periodontal ligament cells [19] and osteoblasts [17]. HERS expresses AMBN, whereas other enamel proteins including amelogenin, enamelin, and tufterin are eliminated in the tooth root developmental process [1]. It may be assumed that AMBN is related to tooth root formation, but its role in root formation remains unclear. To elucidate the role of AMBN in root formation, we investigated the influence of AMBN downregulation on HERS using small interfering RNA (siRNA) for AMBN.

## Materials and Methods

### Immuno-cyotochemical Analyses for Root Developmental Processes

C57BL/6 mice (Japan CLEA, Tokyo, Japan) were used throughout this study. Permission for all experiments in this study was granted by the Animal Experiment Committee of Hiroshima University. Mandibles were dissected and immersed in 4% paraformaldehyde (PFA) in 0.067 M phosphate buffer, pH7.4 at 4°C for 24 h and decalcified with 10% ethylenediaminetetraacetic acid (EDTA) for about 1 week at 4°C. Specimens were embedded in paraffin and cut into sections 5 µm thick along the mesiodistal direction. These sections were mounted on MAS-GP coated glass slides (Matsunami, Osaka, Japan) and stained with hematoxylin (Sigma-Aldrich, St. Louis, MO) and eosin (Sigma-Aldrich).

Deparaffinized sections were rehydrated in 0.01 M phosphate buffered saline (PBS) of pH 7.4. Specimens were dipped in 0.3% H_2_O_2_ for 30 min to block endogenous peroxidase, and then incubated with PBS containing 10% FCS for 30 min. Sections were incubated overnight at 4°C with either one of the following three antibodies. Mouse polyclonal antibody against AMBN (Y48) was generated by immunization of rabbits with synthetic peptides (NKAQQPQIKRDAWRF) [4], and used at a dilution of 0.2 µg/ml. Mouse monoclonal antibody against cytokeratin 5 (Covance, Princeton, NJ) was used at a dilution of 1∶500, and mouse monoclonal antibody against 5-bromo-2′-deoxyuridine (BrdU, Convance) was used at a dilution of 1∶500. After rinsing with PBS, sections were incubated with either biotinylated anti rabbit IgG (Vector, Burlingame, CA) for AMBN or biotinylated anti mouse IgG for cytokeratin 5 and BrdU for 30 min at room temperature. Positive reactions were visualized with 3, 3′-diaminobenzidine solution. The experimental protocols were approved by the Animal Care and Use Committee of Hiroshima University.

### Isolation of Hertwig’s Epithelial Root Sheath (HERS) Cells

Postnatal day 10 C57BL/6 mice have no erupted lower molars. First molar crowns were removed from mandibles and HERS was carefully dissected from the apical root under a stereomicroscope (SZX10; Olympus Tokyo, Japan). Tissues were washed with PBS followed by digestion with 0.05% trypsin in 0.53 mM EDTA for 10 min. Isolated HERS cells were placed in culture dishes with MCDB153 medium (Wako, Osaka, Japan) supplemented with 10% fetal bovine serum (FCS, Life Technologies, Carlsbad, CA), 100 U/ml penicillin, and 100 µg/ml streptomycin (Sigma-Aldrich). The medium was changed every 3 days and cells were cultured at 37°C under 5% CO_2_ in air for 2 weeks. No contaminated fibroblasts survived in this condition due to the low calcium content of MCDB153 medium. For immuno-staining of cultured cells, cells were fixed in PBS containing 4% PFA and 0.5% Triton X-100 for 5 min, and then immuno-staining was performed.

### Total RNA Isolation and Reverse Transcription Polymerase Chain Reaction (PCR)

Total RNA was extracted using RNeasy (Qiagen, Venlo, Netherlands) according to the manufacturer’s instructions. Total RNA of 1.0 µg was reverse-transcribed in 50 mM Tris-HCl (pH 7.4), 75 mM KCl, and 2.5 mM MgCl_2_, 25 µg/ml oligo(dT)_12–18_, 200 U of M-MLV reverse transcriptase (Toyobo, Osaka, Japan), 2 mM dNTP, and 10 mM DTT at 37°C for 30 min. PCR was performed on 1 µl of cDNA using the primer pairs in Table 1. Aliquots of PCR products were separated on a 1% agarose gel containing ethidium bromide and visualized with ultraviolet light.

**Table 1:** The primer pairs for RT-PCR.

Primers	Sequence	Accession number	Product size
AMBN	F: GTG CCG GCA TTT CCT CAA CAA CCT G	NM_009664	349 bp
	R: CTG CAA GGG CAG CTG TCC		
GAPDH	F: AAC TTT GGC ATT GTG GAA GG	NM_008084	517 bp
	R: GGG TTT CTT ACT CCT TGG AG		
p21^Cip1^	F: GAG AAC GGT GGA ACT TTG ACT T	NM_001111099	480 bp
	R: CTC AGA CAC CAG AGT GCA AGA C		
p27^Kip1^	F: GAT ATG GAA GAA GCG AGT CAG C	MMU10440	224 bp
	R: GAG TTT GCC TGA GAC CCA ATT A		
CDK1	F: GGT ACT TAC GGT GTG GTG TAT AAG G	NM_007659	246 bp
	R: GAT GGA GTC CAG GTA CTT CTT GAG		
CDK4	F: GGT CAC CCT AGT GTT TGA GCA TAT AG	NM_009870	207 bp
	R: GTA GAT TCT AGC TAG GCC AAA GTC AG		
CDK6	F: GAC GGA CAG AGA AAC CAA GCT TAC	NM_009873	176 bp
	R: GAT CAC GAT GCA CTA CTC TGT GAG		

### Knockdown of AMBN Expression by Small Interfering RNA (siRNA)

A siRNA targeting AMBN was designed based on the mouse sequence and synthesized by Integrated DNA Technologies (Coralville, IA). The siRNA compounds were 27mer Dicer-substrate duplexes optimized for Dicer processing and showed increased potency when compared with 21mer duplexes. The siRNA oligonucleotides used in this study were as follows; AMBN siRNA: 5′-AGAGCACUAAGCAUAUAUU AAUAAA-3′.

HERS cells were transfected with 40 nM siRNA using Lipofectamine™ RNAiMAX (Life Technologies) according to the manufacturer’s instructions. Forty-eight hours after transfection, total RNA was isolated and RT-PCR was performed to verify the knockdown of AMBN expression.

For injecting siRNA into dental lamina of postnatal day 10 mice, an ultrafinest 33G needle (NanoPass33, Termo, Tokyo, Japan), Hamilton syringe (Hamilton, Reno, NV), and 0.61 mm polyethylene tube (Becton Dickinson, Franklin Lakes, NJ) were used. Under SZX10 (Olympus), either 1 µl AMBN or negative control siRNA solution was injected into the mesial hillside of the mandibular first molar in postnatal day 10 mice. Ten days after the injection, histological observation was performed for the distal root of the first molar because the mesial root may have been affected by surgical or physical damage.

### Cell Preparation for Transient Expression of AMBN

ALC cells had oral epithelial origins and were established from tooth germs of newborn C57BL/6 mice [20]. These cells were maintained in spinner modified minimum essential medium (Sigma-Aldrich) supplemented with 10% FCS (Life Technologies), 100 U/ml penicillin, and 100 µg/ml streptomycin (Sigma-Aldrich) at 37°C under 5% CO_2_ in air. Full length AMBN in the pcDNA3.1 plasmid vector (Life Technologies) or only the vector was transfected into cells using Lipofectamine 2000 (Life Technologies) according to the manufacturer’s instructions. Forty-eight hours after transfection, transient expression of AMBN was confirmed with RT-PCR analyses.

### Statistical Analysis

All the experiments were carried out in triplicate. Results were expressed as means and standard deviations (SD). Student’s t-test was performed to examine significant difference between treated and untreated groups. SSPS software, version 18 (IBM, Armonk, NY) was used for all statistical analyses. Differences between means were considered significantly different when values of *p<0.05 and ** p<0.01.

## Results

**Figure 1:**
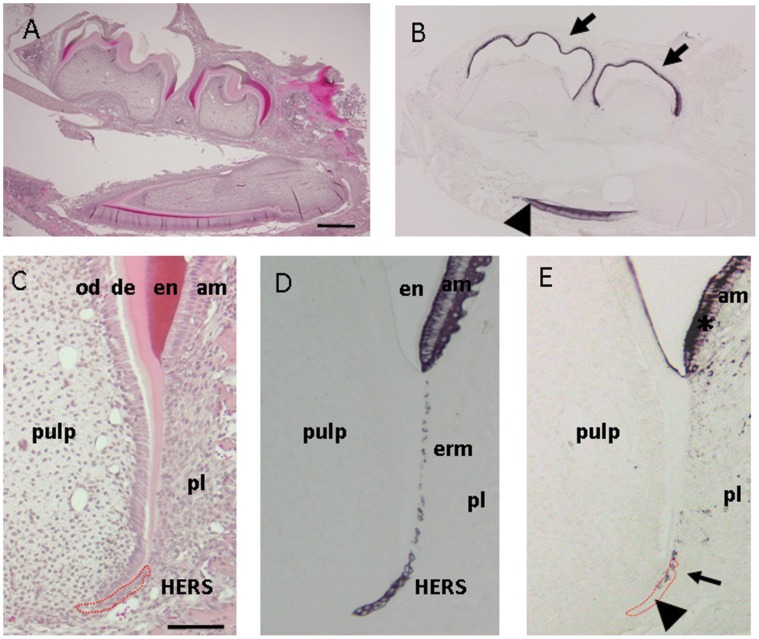
Light micrographs illustrating root development and Hertwig’s epithelial root sheath (HERS) in postnatal day 15 mice. (A) Hematoxylin and eosin staining of the mandible. (B) Immuno-staining with ameloblastin (AMBN). AMBN is localized in ameloblasts of the outer enamel epithelium in the first and the second molars (arrow) and incisor (arrow-head). (C) Hematoxylin and eosin staining of the distal root of the lower first molar. (D) Immuno-staining with cytokeratin 5. Ameloblasts, epithelial cell rests of Malassez, and HERS positively reacted against cytokeratin 5 immunohistochemistry. (E) Immuno-staining with AMBN. Ameloblasts around enamel (asterisk) and in the basal portion of HERS (arrow) reacted with AMBN antibody, but not in the tip (arrowhead). am, ameloblasts; od, odontoblasts; en, enamel; de, dentin; pl, periodontal ligament; erm, epithelial call rests of Malassez; HERS, Hertwig’s root sheath. Red circle marks the location of HERS. Scale bars: 500 µm in (A) and (B), and 100 µm in (C) – (E).

To determine the presence and distribution of AMBN during tooth root developmental processes, we first assessed expression of AMBN in postnatal day 15 mice. As shown in Fig. 1, AMBN was immuno-localized in ameloblasts in the first and second molars and incisors in the mandible. In postnatal day 15 mice, the process of root formation had already been initiated and HERS extended along with dental follicular tissue for root formation. Under higher magnification, HERS was confirmed by immuno-reactivity against cytokeratin 5, indicating their epithelial origin (Fig. 1D), whereas AMBN was immuno-distributed in the basal portion of HERS, not at the tip (Fig. 1E).

**Figure 2:**
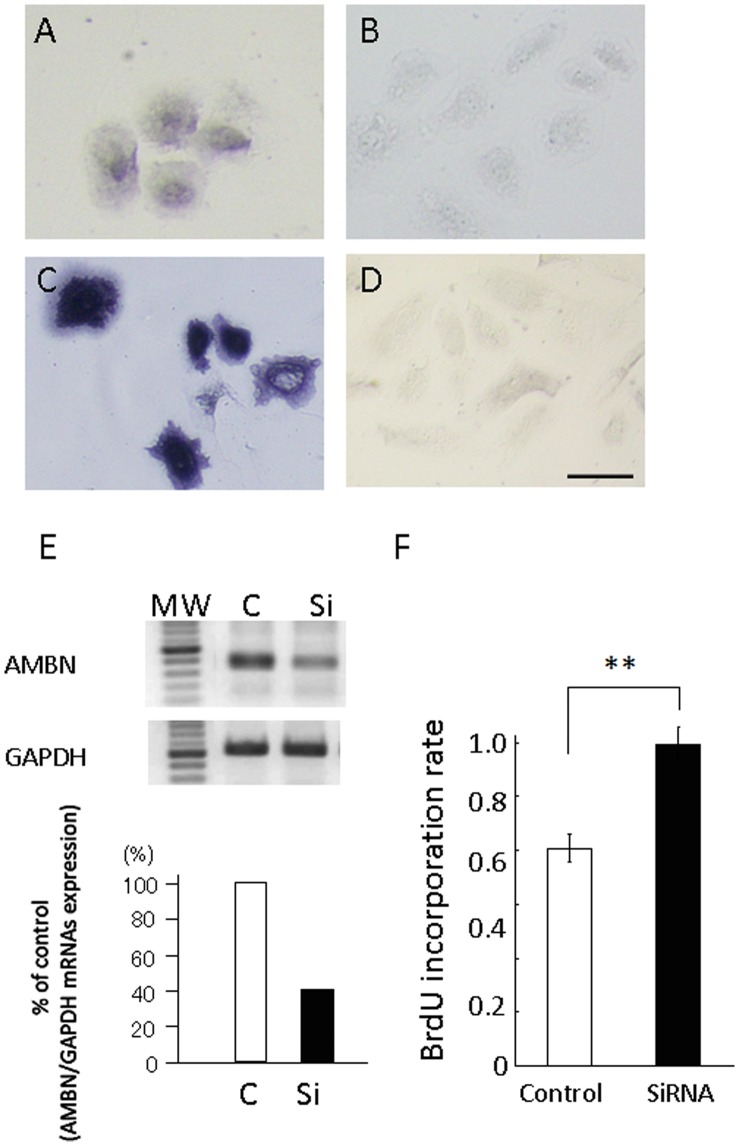
Expression of AMBN in HERS derived cells and suppression of AMBN expression by treatment with AMBN siRNA. HERS derived cells were isolated from postnatal day 10 mice and maintained in MCDB153 medium. (A) Immuno-staining with AMBN. (B) Immuno-staining without AMBN. (C) Immuno-staining with cytokeratin 5. (D) Immuno-staining without cytokeratin 5. Scale bars: 10 µm. (E) Expression of AMBN was analyzed with total RNA from cells treated with either AMBN siRNA or control siRNA. MW; 100 bp molecular weight marker; C, treated with control siRNA; Si, treated with AMBN siRNA. Expression of AMBN was inhibited by 60% when treated with AMBN siRNA. (F) The BrdU incorporation rate of control siRNA group was higher than that of the siRNA treated group two days after siRNA treatment. Statistical analysis was performed using Student’s t-test, **; p<0.01.

To assess the functional roles of AMBN localized in HERS in root development, siRNA targeting AMBN mRNA was designed based on the mouse sequence and HERS cells were isolated from postnatal day 10 mice. These cells were maintained in MCDB153 medium and no contaminated fibroblasts survived in this condition due to the low calcium content. These cells exhibited typical epithelial morphology and revealed reactivity against AMBN (Fig.2A) and cytokeratin 5 (Fig. 2C); however, proliferation was very slow without any confluence. These cells expressed both AMBN mRNA and protein confirmed by RT-PCR analysis and immuno-staining. Treatment with siRNA showed a 60% partial knockdown of AMBN mRNA in HERS cells over that of negative control siRNA (Fig.2E). In addition, treatment with siRNA exhibited an increase in the number of incorporated BrdU in these cells (Fig.2F).

**Figure 3:**
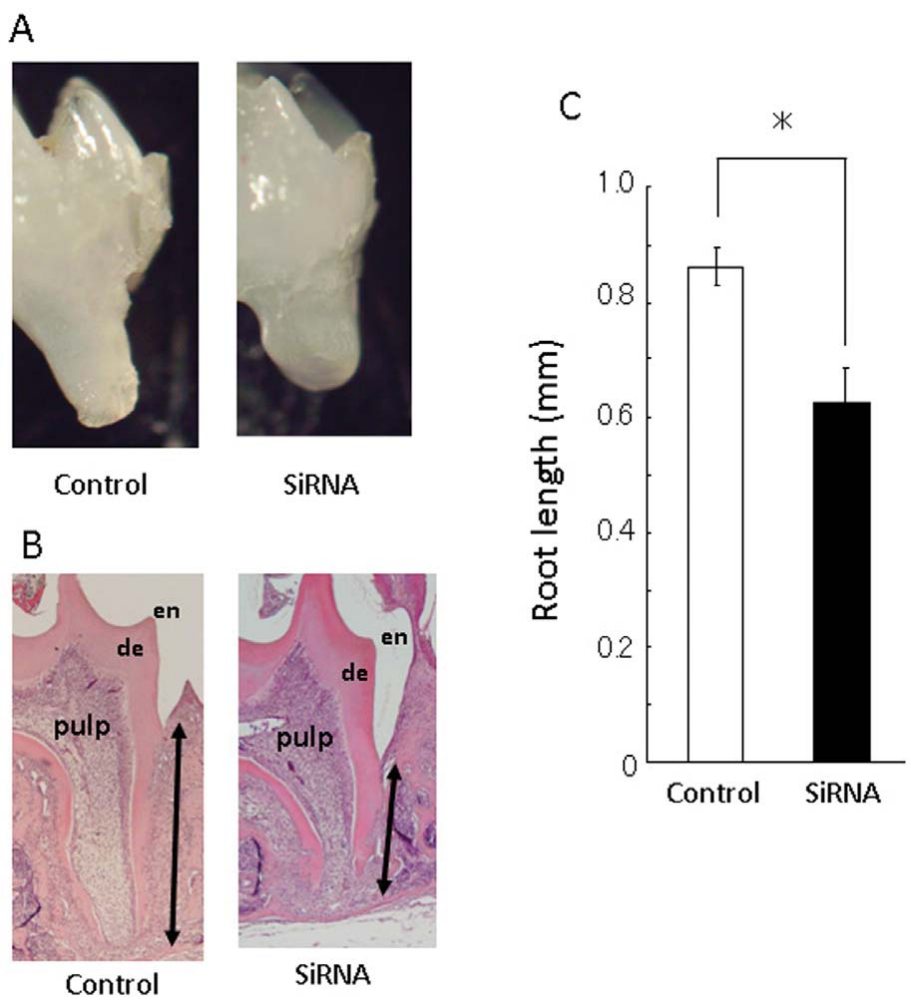
Histological observation of the lower first molar treated with siRNAs. Distal root length of the lower first molar in AMBN siRNA treated was shorter than that of controls. (A) Stereomicroscopic appearance of the lower first molar treated with AMBN siRNA. (B) Hematoxylin and eosin staining in control and AMBN siRNA. Treatment with AMBN siRNA caused irregularities in dentin form at the tip of the root. (C) Average root length in control and AMBN siRNA treated groups. Root length in treatment with AMBN siRNA (n = 10) was shorter than that of control siRNA (n = 10) (Two headed arrow indicates root length in Figure B.). Statistical analysis was performed using Student’s t-test, *; p<0.05.

**Figure 4:**
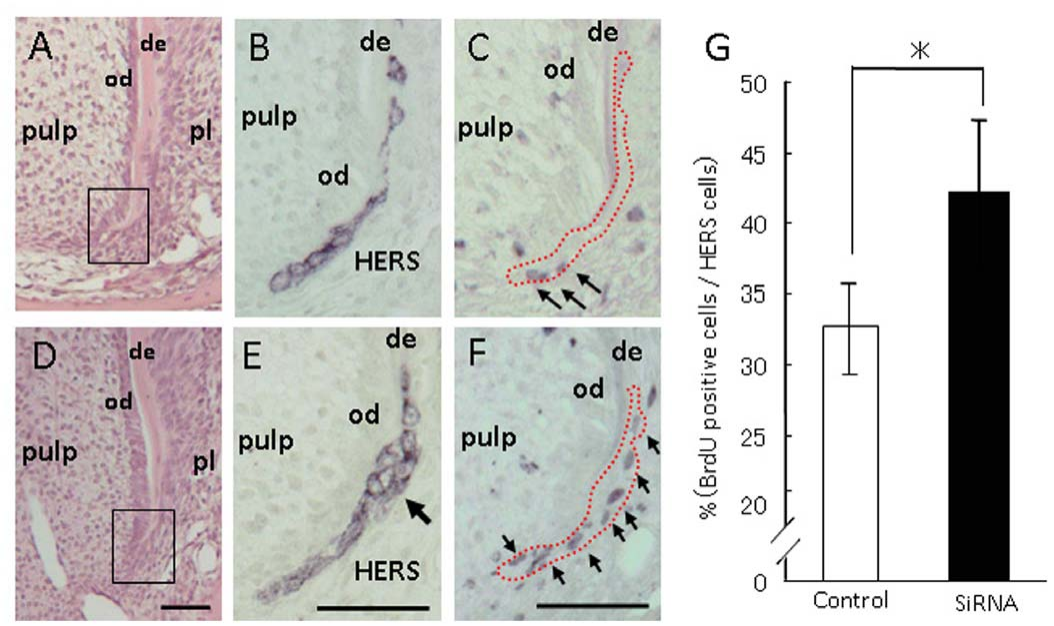
Light micrographs illustrating HERS treated with siRNAs. AMBN siRNA injected mice revealed a multilayered appearance in the basal portion of HERS and increased BrdU positive cells in that of the outer layer. Treatment with control siRNA (A–C) and AMBN siRNA (D–F). (A) and (D) Hematoxylin-eosin staining. (B) and (E) Immuno-staining with cytokeratin 5. Treatment with AMBN siRNA revealed a multilayered appearance in the basal portion of HERS (arrow). (C) and (F) Immuno-staining with AMBN. Treatment with AMBN siRNA increased BrdU positive cells in the outer layer of HERS (arrows). de, dentin; od, odontoblasts; pl, periodontal ligament; HERS, Hertwig’s root sheath. Red dotted lines mark the location of HERS. Scale bars: 100 µm. (G) The percentage of BrdU positive cells per total HERS cells was counted and compared between treatments with control and AMBN siRNA. The percentage of BrdU positive cells was higher with treatment with AMBN siRNA (n = 10) than that with control siRNA (n = 10). Statistical analysis was performed using Student’s t-test, *; p<0.05.

Ten days after the AMBN siRNA injection into dental lamina of postnatal day 10 mice, histological observation was performed in the distal region of the first molar root in order to avoid side effects from surgical damage. AMBN siRNA injected mice displayed shorter roots in the first molar. In addition, these mice presented slightly irregular root dentin structures, as observed histologically (Fig. 3). However, no inflammatory disorder, infiltration of immune cells, or root resorption was observed in the surrounding area of dentin and cementum layers. Under higher magnification in HERS, AMBN siRNA injected mice revealed a multilayered appearance in the basal portion of HERS; although control siRNA injected mice showed two-layered HERS derived from inner and outer enamel epithelia (Fig. 4). Furthermore, AMBN siRNA injected mice revealed that BrdU positive cells increased in number in the outer layer of HERS. This result is consistent with the mitotic index which was significantly higher than in control siRNA mice.

**Figure 5:**
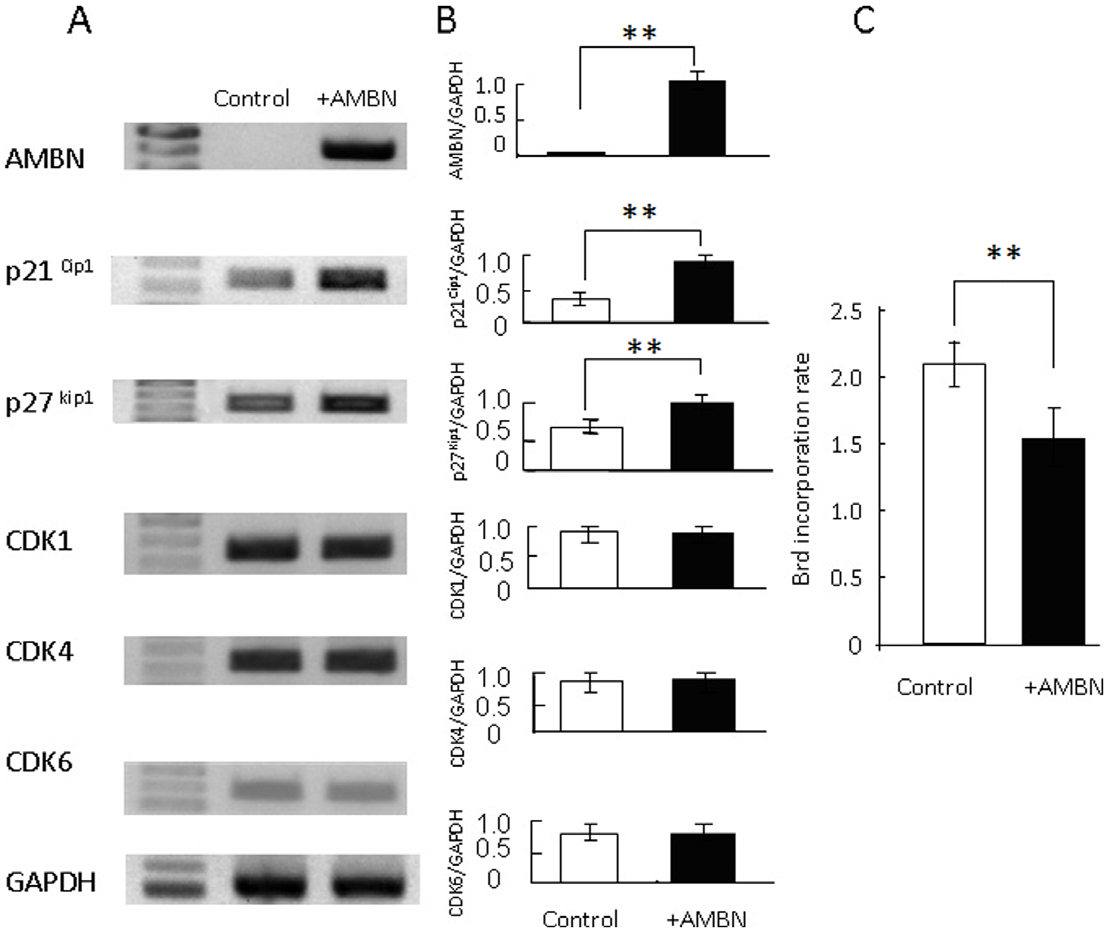
Comparison between AMBN expressing and non expressing cells. Full length AMBN cDNA transfected cells expressed AMBN at a high intensity whereas control cells had no AMBN mRNA. (A) and (B) Positive cell cycle regulatory factors, CDK1, CDK4 and CDK6, and negative cell cycle regulatory factors, p21^Cip1^ and p27^Kip1^, were examined by RT-PCR analysis. AMBN non expressing cells had lower expression levels of negative cell cycle regulators than those of AMBN expressing cells. (C) BrdU positive cells were counted and compared between AMBN expressing and non-expressing cells. Cell numbers in the AMBN non-expressing group was lower than those of the AMBN expressing group. Statistical analysis was performed using Student’s t-test, **; p<0.01.

To examine increases in proliferation by the knockdown of AMBN, we prepared cells transiently expressing AMBN mRNA. These cells expressed AMBN at a high intensity whereas control cells had no AMBN mRNA (Fig. 5). Furthermore, these AMBN non-expressing cells were higher in ratio to BrdU incorporation than AMBN expressing cells. In addition, control cells exhibited weak expression levels in negative cell cycle regulatory factors, such as p21^Cip1^ and p27^Kip1^, although AMBN expressing cells expressed distinct signals for these negative factors. However, there were no significant differences in expression of positive cell cycle regulatory factors, including CDK1, CDK4, and CDK6, between cells with and without AMBN. These results indicate that elimination of AMBN promotes proliferation via inhibition of negative cell cycle regulators.

## Discussion

Ameloblasts secrete an abundance of enamel matrix proteins such as AMBN, amelogenin, enamelin, and tuftelin [20], [21]. Various studies have been conducted to investigate the role of AMBN in enamel formation. AMBN is abundantly present in immature enamel during the matrix formation stage [5], [8], and is identified as a protein with different nucleic acid sequences from other enamel proteins [4]. AMBN has the distinctive function to immediately cleave itself into several fragments after being secreted from ameloblasts [8]. These fragments spread and localize to various sites of newly formed enamel during crown morphogenesis. The short fragment from the n-terminal domain distributes in whole enamel and gradually degrades from the enamel matrix during the matrix formation stage, whereas that from the c-terminal domain distributes in only the superficial layer of enamel and then rapidly degrades from immature enamel. AMBN may be related to formation and maturation of enamel crystallization. In recent studies using knockout mice against enamelin [22], enamelin mutations caused ameloblast malfunctions in cell morphogenesis, detachment from the tooth surface, apoptosis, and formation of ectopic calcifications. In contrast, mutating AMBN, amelogenin, or both in mice caused enamel hyperplasia, with no enamel defects irrespective of disordered functions in ameloblasts [23]. It may thus be considered that these enamel related molecules play important roles in mineral deposition and crystallization in the organization and regulation of enamel formation.

However, a number of observations indicate that AMBN is not an enamel specific protein because AMBN exists in a wide variety of cells including osteoblasts [16], [17], odontoblasts [14], [15], and cementoblasts [18]. AMBN may play an important role in dentin and cementum formation during differentiation processes in the periodontium. HERS extends along with dental follicular tissue and AMBN is highly distributed in the basal areas of HERS. A histological study showed that ameloblasts change expression levels of AMBN and amelogenin during enamel formation [24]. Based on this evidence, constituted cells in HERS may modulate expression levels of AMBN in a developing and/or site specific manner for root development similar to enamel formation.

This study was designed to focus on root formation, unintended consequences which eliminated AMBN in enamel formation were undesired. Injection of siRNA into cells provided useful information about the undefined functions and roles of various gene molecules at a specific stage [25], [26]. A method involving siRNA in vitro is a more convenient and inexpensive technique than that with knockout mutants. However, intravenous injection of siRNA in vivo has disadvantages in cost and effect because it is hard to deliver siRNA into target organs through the circulation and it is necessary to inject large amounts of siRNA. On the other hand, local administration of siRNA into living organisms has been shown to be beneficial, but it is necessary to apply to a confined space in order to avoid leaking reagent solution over a long period, such as lumbar vertebra, retina, and the brain [27].

In this study, microinjection of AMBN siRNA into the mesial side of a prospective mandibular first molar was performed in postnatal day 10 mice. Ten-day-old mice have no erupted and developing molars in jaws and their molars are surrounded by alveolar bone with a confined space where reagent solution can be kept. In local administration, AMBN siRNA is reliably able to reach the tip of the tooth root. AMBN siRNA injection revealed shorter roots and slightly irregular root dentin formation in mice and HERS cells showed abnormal proliferation. Meanwhile, a previous study using AMBN knockout mice demonstrated an influence on enamel formation, but not root formation [11], [12]. It has recently been shown that previously-believed AMBN knockout mice still produce a short truncated form of AMBN, although this is not the full length of AMBN [13]. Such a short truncated form of AMBN still contains the binding domains for calcium, fibronectin, and heparin. The truncated form of AMBN influences enamel formation, but quantitative depletion of AMBN in this study may be important for root formation. Elimination of AMBN may cause modulation of the differentiation status of HERS cells as well as proliferation. A disparity in AMBN expression in HERS may result in the modulation of reciprocal influences to the peripheral local environment including odontoblasts.

A number of genes are involved in cell division regarding positive and negative cell cycle regulatory factors. CDK1, CDK4, and CDK6 are positive regulators, and p21^Cip1^ and p27^Kip1^ are negative regulators in the cell cycle [11]. In this study, there was a considerable discrepancy between cells expressing AMBN and their proliferation. Omitting AMBN in cells diminished the inhibitory effects of p21^Cip1^ and p27^Kip1^, thereby cells increased their ratio of BrdU incorporation. A previous study revealed that overexpression of AMBN in human ameloblastoma inhibited proliferation through suppression of negative cell cycle regulators including p21^Cip1^ and p27^Kip1^ [11]. However, overexpression of AMBN with a defect in heparin binding domains had relatively little effect on proliferation. Therefore, heparin binding domains in AMBN may be important for regulating cell growth. From these findings, it may be presumed that suitable expression of AMBN in HERS may function as a certain trigger of root formation and the progress essential for optimal root development.
